# An atypical case of adenomyosis coexisting with simple endometrial hyperplasia: A case report

**DOI:** 10.1097/MD.0000000000048940

**Published:** 2026-05-15

**Authors:** Xiaonan Ma, Ruiyi Tang, Rong Chen

**Affiliations:** a National Clinical Research Center for Women’s Health and Obstetric and Gynecologic Diseases, Department of Obstetrics and Gynecology, Peking Union Medical College Hospital, Chinese Academy of Medical Sciences & Peking Union Medical College, Beijing, China.

**Keywords:** adenomyomatous polyps, adenomyosis, atypical presentation, endometrial hyperplasia, fertility preservation

## Abstract

**Rationale:**

Adenomyosis and simple endometrial hyperplasia are common benign gynecological conditions. However, atypical presentations with extreme endometrial thickening and associated diagnostic challenges have rarely been reported.

**Patient concerns:**

A 27-year-old woman presented with a 13-year history of menstrual irregularity and endometrial thickness of 7.0 cm on ultrasonography. Magnetic resonance imaging (MRI) revealed an enlarged uterus and a 7.9 cm intrauterine mass (T1 isointense, T2 hyperintense, diffusion-weighted imaging high signal) with junctional zone interruption.

**Interventions:**

Taking into account the patient’s reproductive needs and the fact that hysteroscopy couldn’t completely remove the contents of the uterine cavity, an open surgery was used for diagnosis and treatment.

**Diagnoses:**

Pathological examination led to the diagnosis of adenomyosis combined with non-atypical endometrial hyperplasia.

**Outcomes:**

After 6 months of postoperative gonadotropin-releasing hormone agonist (GnRH-a) treatment, the patient achieved regular menstruation. During 10 years of follow-up, she had 2 natural pregnancies and delivered 2 full-term infants via cesarean section.

**Lessons:**

This case highlights that adenomyosis can present with an extremely thickened endometrium, an intrauterine mass and the absence of typical dysmenorrhea. Combined surgery and GnRH-a can achieve long-term symptom control and successful pregnancies, supporting fertility-preserving management in similar young patients.

## 1. Introduction

Adenomyosis is a common gynecological disorder characterized by invasion of endometrial tissues (glands and stroma) into the myometrium. The symptoms include prolonged or heavy menstrual bleeding and progressive dysmenorrhea. Adenomyosis can be diagnosed with reasonable accuracy using ultrasonography and magnetic resonance imaging (MRI). Classic MRI findings include a punctate high signal within the myometrium, maximum junctional zone (JZ) thickness > 12 mm, and a ratio of maximal JZ thickness to myometrial thickness > 40%.^[1]^ In recent years, MRI features of uterine adenomyosis have been used to classify this disease.

Endometrial hyperplasia (EH) is characterized by an increased number of endometrial glands with varying sizes and irregular shapes. According to the 2020 World Health Organization (WHO) classification, EH is categorized as non-atypical hyperplasia (which includes the former simple and complex types) or atypical hyperplasia.^[2]^ Clinical manifestations include abnormal uterine bleeding and risk of progression to carcinoma. Long-term estrogen stimulation without the antagonistic effects of progesterone is the main cause.^[3]^

Recent studies have reported that adenomyosis and EH frequently coexist, suggesting a possible mechanistic link.^[4,5]^ However, even common diseases may lack typical clinical and pathological features, thereby affecting their diagnosis and treatment. This case is atypical in 3 respects: an endometrial thickness of 7.0 cm (extremely rare), absence of typical dysmenorrhea, and preoperative MRI that did not diagnose adenomyosis despite JZ disruption. The 10-year follow-up of successful pregnancies provides valuable clinical evidence for fertility-preservation management.

## 2. Case presentation

### 2.1. 2002 to 2014: menstrual history before admission

A 27-year-old woman presented to our outpatient clinic on June 24, 2015, with a chief complaint of menstrual irregularity for 13 years and ultrasound-detected endometrial thickening for 6 years. Menarche occurred at 14 years of age (2002). From the onset, her cycles ranged from 1 to 2 months, and bleeding duration varied from 5 days to 1 month. The patient had mild anemia due to prolonged bleeding. Dysmenorrhea was mild and stable over time. In 2009 (age 21 years), the patient underwent ultrasonography for menstrual disorders, and the thickness of the endometrium was 3.4 cm. Traditional Chinese medicine was ineffective, and menstrual irregularities and endometrial thickening persisted. She had no history of prior surgeries, chronic diseases, endometriosis, or pregnancy.

### 2.2. June 2015: initial evaluation

On admission, a bimanual examination revealed an anteverted uterus at 14 to 15 weeks of gestation with firm consistency, and no tenderness. Laboratory examinations revealed mild iron-deficiency anemia (hemoglobin 98 g/L). The sex hormone levels on day 5 of the menstrual cycle were as follows: follicle-stimulating hormone (FSH), 2.30 IU/L; luteinizing hormone (LH), 3.74 IU/L; estradiol (E2), 166 pg/mL; testosterone, 0.49 ng/mL; and prolactin, 13.30 ng/mL. CA125 was 117.3 U/mL; all other tumor markers were within normal ranges.

Pelvic ultrasound: uterus 12.3 cm × 11.0 cm × 9.0 cm; endometrial thickness approximately 7.0 cm with heterogeneous echo. The myometrial echo was generally uniform without obvious thickening; the adnexa were normal; no masses were observed in the bilateral adnexa.

**Figure 1.**
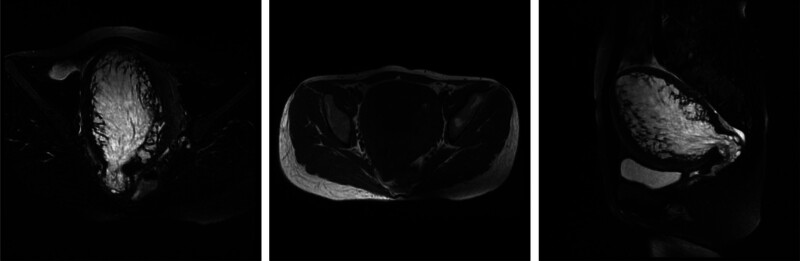
Contrast enhanced MRI of pelvic. MRI = magnetic resonance imaging.

Magnetic resonance imaging (Fig. 1) showed an enlarged uterus with an intrauterine mass (7.9 cm) showing T1 isointensity, T2 hyperintensity, and irregular margins. Diffusion-weighted imaging (DWI) showed high signal intensity. The junctional zone (JZ) was interrupted. The anterior myometrial thickness was 17.4 mm, and the posterior 10.3 mm. No microcysts or clear junctional zone–myometrium interface was observed anteriorly.

### 2.3. June 2015 (first procedure): hysteroscopy

**Figure 2.**
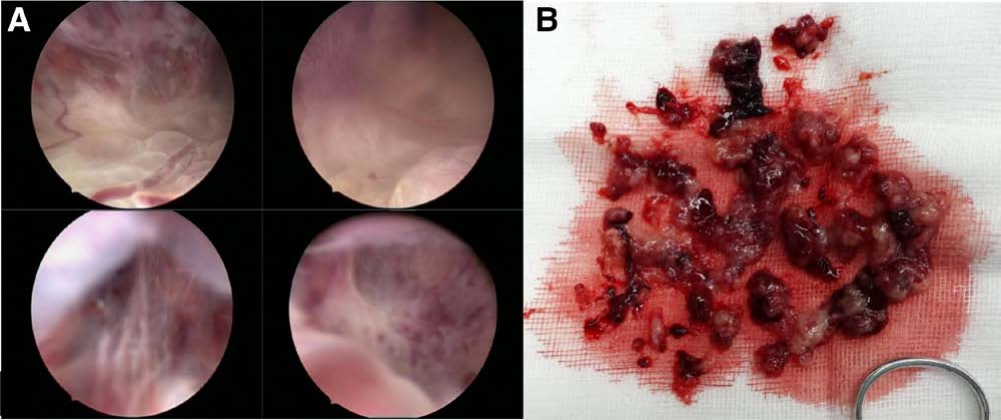
(A) Hysteroscopy surgery photos. (B) Contents scraped from the uterine cavity.

Hysteroscopy was performed for the diagnosis. The uterine cavity was occupied by a mass > 15 cm deep, and the fundus could not be fully probed. The mass had an abundant blood supply and was partially solid, compartmentalized, and filled with vesicular tissue (Fig. 2A). Normal uterine parietal tissue was not observed. Curettage specimens (Fig. 2B) were sent to frozen sections, which revealed endometrial polyps and interstitial edema. The intrauterine tumor mass was removed using an oval clamp, and necrotic tissue was aspirated using negative pressure. However, after partial resection, the uterine cavity remained filled with these substances. Considering the patient’s desire for pregnancy and the fact that hysteroscopy could not solve the problem of the uterine cavity contents, after discussions with the patient’s family, we decided to wait for the pathological results before further treatment. Paraffin histopathology suggested the presence of endometrial polyps and a simple hyperplastic endometrium.

### 2.4. July 2015 (second procedure): open surgery and wedge resection

Given the failure of hysteroscopy to resolve the excessive contents in the uterine cavity and the patient’s strong desire for future fertility, we proceeded with open surgery 2 weeks later. Intraoperatively, the myometrium was markedly thickened. After a longitudinal incision, the uterine cavity showed a significantly thickened endometrium with edematous tissue, and the endometrium–myometrium interface was indistinct. The endometrial lesions were curetted, and wedge resection of the abnormally thickened uterine muscle layer was performed. The uterine incision was closed using interrupted sutures. Pathological examination confirmed secretory endometrium with interstitial edema and uterine adenomyosis.

Through surgical and pathological verification, the patient was found to have multiple coexisting pathologies: endometrial polyps and simple non-atypical hyperplasia (from hysteroscopy) and adenomyosis (from open surgery). This combination explains the severe uterine enlargement and extreme endometrial thickening and highlights that adenomyosis can coexist with other benign uterine lesions, thereby increasing the difficulty of diagnosis. A large intrauterine mass (7.9 cm) with T2 hyperintensity and a high DWI signal was the dominant imaging finding, leading the radiologist to prioritize endometrial polyps or submucosal fibromas. JZ interruption was noted but was considered secondary to the mass effect rather than primary adenomyosis. In retrospect, this case illustrates that a large intrauterine lesion can mask underlying adenomyosis.

### 2.5. *Postoperative follow-up (2015–2025*)

The patient received 1 dose of gonadotropin-releasing hormone agonist (GnRH-a) immediately after surgery, followed by a total of 5 doses (last dose in December 2015). One year after the open surgery, ultrasound showed a reduced uterine size (7.1 cm × 6.4 cm × 5.8 cm), a single-layer endometrium of 0.25 cm, and uneven myometrial echoes (anterior wall 1.5 cm, posterior 2.9 cm).

**Figure 3.**
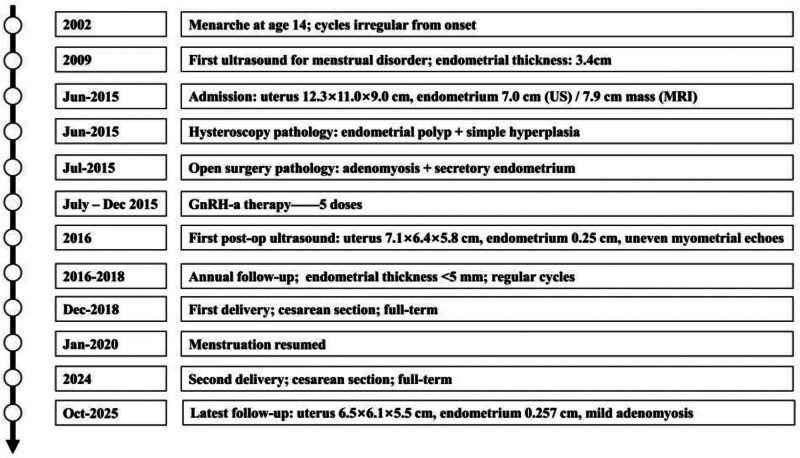
Timeline of clinical course and follow-up. GnRH-a = gonadotropin-releasing hormone agonist, MRI = magnetic resonance imaging, US = ultrasound .

Thereafter, annual ultrasound follow-up was performed. The endometrial thickness remained consistently at <5 mm after each menstruation. Ultrasound showed only mild adenomyosis (anterior wall 1.7 cm, posterior wall 2.6 cm) with no evidence of hyperplasia recurrence or new mass formation (Fig. 3).

Fortunately, the patient conceived naturally and delivered a full-term baby via cesarean section in December 2018. After 9 months of breastfeeding, menstruation resumed spontaneously in January 2020. She conceived naturally again and delivered her second child via cesarean section in 2024. Since then, her menstrual cycles have been regular, with mild bleeding and no dysmenorrhea. The most recent ultrasound (October 2025) showed a uterus of 6.5 cm × 6.1 cm × 5.5 cm, endometrial thickness 0.257 cm, and mild adenomyosis (anterior wall 1.7 cm, posterior wall 2.6 cm).

## 3. Discussion

We report the case of a young woman with adenomyosis coexisting with simple non-atypical endometrial hyperplasia, extremely thickened endometrium, an intrauterine mass (7.0 cm), and the absence of typical dysmenorrhea. A preoperative diagnosis was not made because a large intrauterine mass dominated the imaging findings. The pathogenesis of adenomyosis is relatively complex, with theories encompassing endometrial basal layer defects and tissue damage repair, secondary Müllerian system metaplasia, adult stem cell differentiation, etc.^[6,7]^ Some scholars believe that its pathogenesis is not the result of a single mechanism but a combination of multiple processes.^[8]^

Adenomyosis and endometrial hyperplasia may share a common etiological etiology. Bergholt et al reported that endometrial hyperplasia was the only variable significantly associated with adenomyosis (OR = 3.0, 95% CI: 1.2–8.3) through a retrospective study of 549 cases of hysterectomy.^[9]^ Studies have reported that when the endometrium in adenomyosis is ≥1.3 cm, the risk of EH increases 27 times. Additionally, studies have confirmed that adenomyosis has unique pathophysiological characteristics, including markedly increased estrogen synthesis and slow metabolic processing, leading to substantial estrogen accumulation. Concurrently, decreased expression of progesterone receptors weakens the regulatory effect of progesterone on the endometrium, thereby greatly enhancing tissue growth activity at the interface between the endometrium and myometrium.^[10,11]^ Additionally, the dysregulation of estrogen and progesterone receptor expression in patients with adenomyosis induces abnormal proliferation of the endometrial basal layer, which subsequently triggers pathological endometrial hyperplasia. However, the causality remains unclear: whether the link is driven by a shared high-estrogen milieu (e.g., chronic anovulation) or by adenomyosis itself, which alters endocrine signaling. In our patient, chronic anovulation likely provided unopposed estrogen stimulation, which is a well-known risk factor for both EH and adenomyosis. Thus, the 2 conditions may share a common upstream driver, rather than having a direct causal relationship.

According to the consensus of Chinese experts in the diagnosis and treatment of adenomyosis (2020), it can be classified as diffuse, focal, or specific types including adenomyomatous polyp (AP) and atypical polypoid adenomyoma (APA). AP consists of smooth muscles, endometrial glands, and often scant stroma. Consistent with the findings of the present report, Fitzhugh reported a case of AP with refractory uterine bleeding, revealing a large sessile polypoid mass containing thick-walled vessels, sparse smooth muscle, glands, and stroma.^[12]^ Histopathologically, most AP are CD10-positive (100%) and desmin-positive (97%).^[13]^ One case of exophytic AP mimicked malignancy, penetrating the uterine serosa and forming an ovarian endometrial cyst; after open abdominal resection and postoperative GnRH‑a treatment, the patient achieved satisfactory reproductive outcomes.^[14]^ In retrospect, our case showed some overlap with AP (endometrial glands, stroma, and smooth muscle components). However, definitive classification requires CD10 and desmin immunohistochemistry, which were not performed because of the retrospective nature and limited tissue availability. Therefore, we describe this case as adenomyosis with an atypical presentation rather than a confirmed AP.

Atypical polypoid adenomyoma are rare lesions with uncertain malignant potential and are characterized by glandular atypia and larger smooth muscle components. Approximately 17% to 28% of APA cases are associated with concurrent endometrial adenocarcinoma, and recurrence rates of 24% to 45% have been reported after conservative treatment.^[15-17]^ Our patient showed no glandular atypia in multiple pathological specimens. After 10 years of follow‑up, no evidence of malignant transformation was observed. These findings strongly argue against a diagnosis of APA.

For symptomatic adenomyosis in women desiring fertility, focal adenomyomas can be removed by laparoscopy or laparotomy, followed by postoperative GnRH-a to reduce recurrence.^[18,19]^ Our patient underwent hysteroscopic debulking (incomplete), followed by open-wedge resection of the adenomyotic lesions and 6 months of GnRH-a therapy. This sequential approach resulted in the normalization of uterine size, regular menstruation, and 2 spontaneous pregnancies. The 10-year follow-up confirms the durability of this strategy.

In conclusion, we report a case of uterine adenomyosis combined with simple hyperplasia of the endometrium that is diagnostically perplexing. It can provide experience for similar cases: In young women with long-standing menstrual disorders and extreme endometrial thickening, adenomyosis should be considered even when typical dysmenorrhea is absent and imaging is dominated by a mass lesion. Junctional zone disruption on MRI should not be dismissed as a secondary effect; it may be a clue to the underlying adenomyosis. Combined open-wedge resection of adenomyosis and postoperative GnRH-a can achieve long-term symptom control and successful pregnancy.

There are still limitations in this report. This is was single case report. Immunohistochemistry was not performed, precluding a definitive classification as AP. No systematic comparison with medical therapy alone is available. Despite these limitations, the long follow-up period and reproductive outcomes provided valuable real-world evidence.

## 4. Conclusions

This case demonstrates that adenomyosis can present with an extremely thickened endometrium and an intrauterine mass (7.0 cm) without the typical dysmenorrhea, leading to preoperative diagnostic difficulties. Despite the lack of classical MRI signs, junctional zone disruption should be suspected. A sequential surgical approach (hysteroscopy followed by open wedge resection) combined with postoperative GnRH-a achieved 10-year recurrence-free survival and 2 successful natural pregnancies. For young patients who desire fertility, aggressive fertility-preserving surgery combined with hormone suppression may offer favorable reproductive outcomes.

## Author contributions

**Supervision:** Rong Chen.

**Writing – original draft:** Xiaonan Ma.

**Writing – review & editing:** Rong Chen, Ruiyi Tang.
